# Evaluating Statistical Methods Using Plasmode Data Sets in the Age of Massive Public Databases: An Illustration Using False Discovery Rates

**DOI:** 10.1371/journal.pgen.1000098

**Published:** 2008-06-20

**Authors:** Gary L. Gadbury, Qinfang Xiang, Lin Yang, Stephen Barnes, Grier P. Page, David B. Allison

**Affiliations:** 1 Department of Statistics, Kansas State University, Manhattan, Kansas, United States of America; 2 Department of Mathematics and Statistics, Missouri University of Science and Technology, Rolla, Missouri, United States of America; 3 Department of Biostatistics, School of Public Health, University of Alabama at Birmingham, Birmingham, Alabama, United States of America; 4 Endo Pharmaceuticals, Chadds Ford, Pennsylvania, United States of America; 5 Department of Pharmacology and Toxicology, University of Alabama at Birmingham, Birmingham, Alabama, United States of America; 6 Center for Nutrient–Gene Interaction, University of Alabama at Birmingham, Birmingham, Alabama, United States of America; 7 Clinical Nutrition Research Center, University of Alabama at Birmingham, Birmingham, Alabama, United States of America; The University of Queensland, Australia

## Abstract

Plasmode is a term coined several years ago to describe data sets that are derived from real data but for which some truth is known. Omic techniques, most especially microarray and genomewide association studies, have catalyzed a new zeitgeist of data sharing that is making data and data sets publicly available on an unprecedented scale. Coupling such data resources with a science of plasmode use would allow statistical methodologists to vet proposed techniques empirically (as opposed to only theoretically) and with data that are by definition realistic and representative. We illustrate the technique of empirical statistics by consideration of a common task when analyzing high dimensional data: the simultaneous testing of hundreds or thousands of hypotheses to determine which, if any, show statistical significance warranting follow-on research. The now-common practice of multiple testing in high dimensional experiment (HDE) settings has generated new methods for detecting statistically significant results. Although such methods have heretofore been subject to comparative performance analysis using simulated data, simulating data that realistically reflect data from an actual HDE remains a challenge. We describe a simulation procedure using actual data from an HDE where some truth regarding parameters of interest is known. We use the procedure to compare estimates for the proportion of true null hypotheses, the false discovery rate (FDR), and a local version of FDR obtained from 15 different statistical methods.

## Author Summary

Plasmode is a term used to describe a data set that has been derived from real data but for which some truth is known. Statistical methods that analyze data from high dimensional experiments (HDEs) seek to estimate quantities that are of interest to scientists, such as mean differences in gene expression levels and false discovery rates. The ability of statistical methods to accurately estimate these quantities depends on theoretical derivations or computer simulations. In computer simulations, data for which the true value of a quantity is known are often simulated from statistical models, and the ability of a statistical method to estimate this quantity is evaluated on the simulated data. However, in HDEs there are many possible statistical models to use, and which models appropriately produce data that reflect properties of real data is an open question. We propose the use of plasmodes as one answer to this question. If done carefully, plasmodes can produce data that reflect reality while maintaining the benefits of simulated data. We show one method of generating plasmodes and illustrate their use by comparing the performance of 15 statistical methods for estimating the false discovery rate in data from an HDE.

## Introduction

“Omic” technologies (genomic, proteomic, etc.) have led to high dimensional experiments (HDEs) that simultaneously test thousands of hypotheses. Often these omic experiments are exploratory, and promising discoveries demand follow-up laboratory research. Data from such experiments require new ways of thinking about statistical inference and present new challenges. For example, in microarray experiments an investigator may test thousands of genes aiming to produce a list of promising candidates for differential genetic expression across two or more treatment conditions. The larger the list, the more likely some genes will prove to be false discoveries, i.e. genes not actually affected by the treatment.

Statistical methods often estimate both the proportion of tested genes that are differentially expressed due to a treatment condition and the proportion of false discoveries in a list of genes selected for follow-up research. Because keeping the proportion of false discoveries small ensures that costly follow-on research will yield more fruitful results, investigators should use some statistical method to estimate or control this proportion. However, there is no consensus on which of the many available methods to use [1]. How should an investigator choose?

Although the performance of some statistical methods for analyzing HDE data has been evaluated analytically, many methods are commonly evaluated using computer simulations. An analytical evaluation (i.e., one using mathematical derivations to assess the accuracy of estimates) may require either difficult-to-verify assumptions about a statistical model that generated the data or a resort to asymptotic properties of a method. Moreover, for some methods an analytical evaluation may be mathematically intractable. Although evaluations using computer simulations may overcome the challenge of intractability, most simulation methods still rely on the assumptions inherent in the statistical models that generated the data. Whether these models accurately reflect reality is an open question, as is how to determine appropriate parameters for the model, what realistic “effect sizes” to incorporate in selected tests, as well as if and how to incorporate correlation structure among the many thousands of observations per unit [2].

Plasmode data sets may help overcome the methodological challenges inherent in generating realistic simulated data sets. Catell and Jaspers [3] made early use of the term when they defined a plasmode as “a set of numerical values fitting a mathematico-theoretical model. That it fits the model may be known either because simulated data is produced mathematically to fit the functions, or because we have a real—usually mechanical—situation which we know with certainty must produce data of that kind.” Mehta et al. (p. 946) [2] more concisely refer to a plasmode as “a real data set whose true structure is known.” The plasmodes can accommodate unknown correlation structures among genes, unknown distributions of effects among differentially expressed genes, an unknown null distribution of gene expression data, and other aspects that are difficult to model using theoretical distributions. Not surprisingly, the use of plasmode data sets is gaining traction as a technique of simulating reality-based data from HDEs [4].

A plasmode data set can be constructed by spiking specific mRNAs into a real microarray data set [5]. Evaluating whether a particular method correctly detects the spiked mRNAs provides information about the method's ability to detect gene expression. A plasmode data set can also be constructed by using a current data set as a template for simulating new data sets for which some truth is known. Although in early microarray experiments, sample sizes were too small (often only 2 or 3 arrays per treatment condition) to use as a basis for a population model for simulating data sets, larger HDE data sets have recently become publicly available, making their use feasible for simulation experiments.

In this paper, we propose a technique to simulate plasmode data sets from previously produced data. The source-data experiment was conducted at the Center for Nutrient–Gene Interaction (CNGI, www.uab.edu/cngi), at the University of Alabama at Birmingham. We use a data set from this experiment as a template for producing a plasmode null data set, and we use the distribution of effect sizes from the experiment to select expression levels for differentially expressed genes. The technique is intuitively appealing, relatively straightforward to implement, and can be adapted to HDEs in contexts other than microarray experiments. We illustrate the value of plasmodes by comparing 15 different statistical methods for estimating quantities of interest in a microarray experiment, namely the proportion of true nulls (hereafter denoted π_0_), the false discovery rate (FDR) [6] and a local version of FDR (LFDR) [7]. This type of analysis enables us, for the first time, to compare key omics research tools according to their performance in data that, by definition, are realistic exemplars of the types of data biologists will encounter. The illustrations given here provide some insight into the relative performance characteristics of the 15 methods in some circumstances, but definitive claims regarding uniform superiority of one method over another would require more extensive evaluations over multiple types of data sets.

## Results

### Simulation Design – Producing the Plasmode Data Sets

Steps for plasmode creation that are described herein are relatively straightforward. First, an HDE data set is obtained that reflects the type of experiment for which statistical methods will be used to estimate quantities of interest. Data from a rat microarray experiment at CNGI were used here. Other organisms might produce data with different structural characteristics and methods may perform differently on such data. The CNGI data were obtained from an experiment that used rats to test the pathways and mechanisms of action of certain phytoestrogens [8],[9]. In brief, rats were divided into two large groups, the first sacrificed at day 21 (typically the day of weaning for rats), the second sacrificed at day 50 (the day, corresponding to late human puberty, when rats are most susceptible to chemically induced breast cancer). Each of these groups was subdivided into smaller groups according to diet. At 21 and 50 days, respectively, the relevant tissues from these rat groups were appropriately processed, and gene expression levels were extracted using GCOS (GeneChip Operating Software). We exported the microarray image (*.CEL) files from GCOS and analyzed them with the Affymetrix Package of Bioconductor/R to extract the MAS 5.0 processed expression intensities. The arrays and data were investigated for outliers using Pearson's correlation, spatial artifacts [10] and a deleted residuals approach [11]. It is important to note that only one normalization method was considered, but the methods could be compared on RMA normalized data as well. In fact, comparisons of methods' performances on data from different normalization techniques could be done using the plasmode technique.

**Figure 1:**
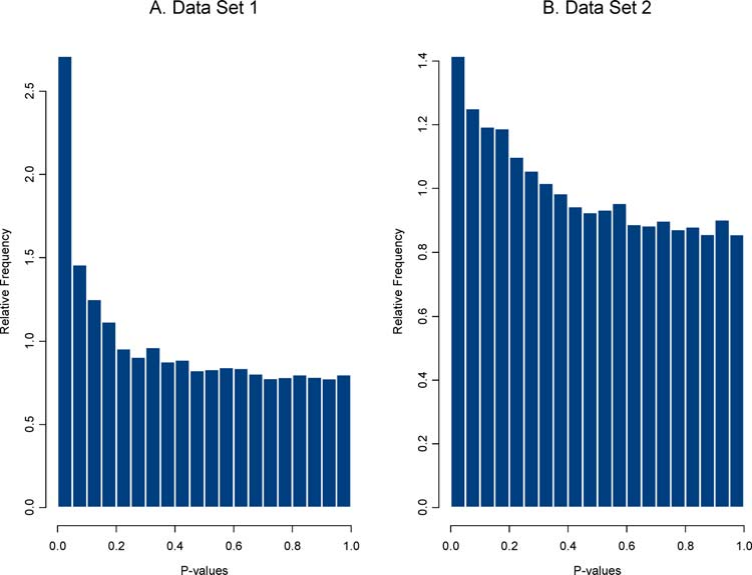
Distribution of P-values from tests for differential expression for the two data sets. P-values were computed from the original data using two sample pooled variance t-tests.

Second, an HDE data set that compares effect of a treatment(s) is analyzed and the vector of effect sizes is saved. The effect size used here was a simple standardized mean difference (i.e., a two sample t-statistics) but any meaningful metric could be used. Plasmodes, in fact, could be used to compare the performance of statistical methods when different statistical tests were used to produce the P-values. We chose two sets of HDE data as templates to represent two distributions of effect sizes and two different null distributions. We refer to the 21-day experiment using the control group (8 arrays) and the treatment group (EGCG supplementation, 10 arrays) as data set 1, and the 50-day experiment using the control group (10 arrays) and the treatment group (Resveratrol supplementation, 10 arrays) as data set 2. There were 31,042 genes on each array, and two sample pooled variance t-tests for differential expression were used to create a distribution of P-values. Histograms of the distributions for both data sets are shown in Figure 1.

The distribution of P-values for data set 1 shows a stronger signal (i.e., a larger collection of very small P-values) than that for data set 2, suggesting either that more genes are differentially expressed or that those that are expressed have a larger magnitude treatment effect. This second step provided a distribution of effects sizes from each data set.

**Figure 2:**
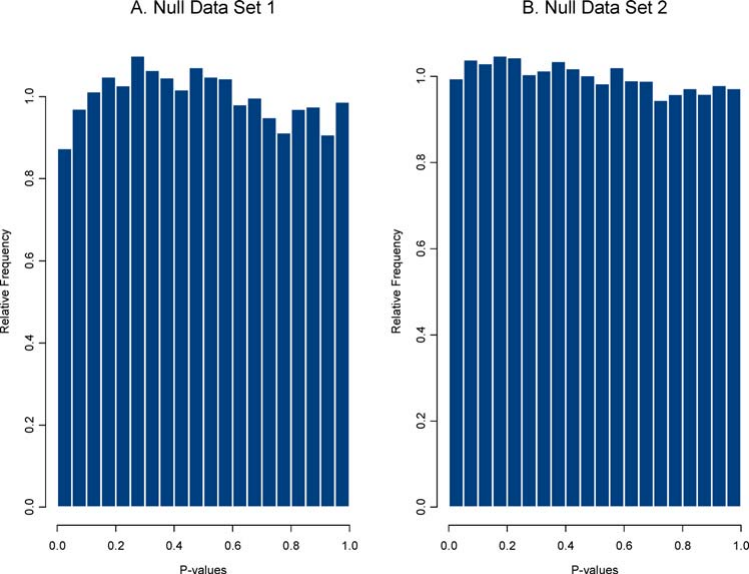
Distribution of P-values for the two plasmode null data sets. P-values were computed from two sample pooled variance t-tests.

Next, create the plasmode null data set. For each of the HDE data sets, we created a random division of the control group of microarrays into two sets of equal size. One consideration in doing so is that if some arrays in the control group are ‘different’ from others due to some artifact in the experiment, then the null data set can be sensitive to how the arrays are divided into two sets. Such artifacts can be present in data from actual HDEs, so this issue is not a limitation of plasmode use but rather an attribute of it, that is, plasmodes are designed to reflect actual structure (including artifacts) in a real data set. We obtained the plasmode null data set from data set 1 by dividing the day 21 control group of 8 arrays into two sets of 4, and for data set 2 by dividing the control group of 10 arrays into two sets of 5 arrays. Figure 2 shows the two null distributions of P-values obtained using the two sample t-test on the plasmode null data sets. Both null distributions are, as expected, approximately uniform, but sampling variability allows for some deviation from uniformity.

A proportion 1−π_0_ of effect sizes were then sampled from their respective distributions using a weighted probability sampling technique described in the Methods section. What sampling probabilities are chosen can be a tuning parameter in the plasmode creation procedure. The selected effects were incorporated into the associated null distribution for a randomly selected proportion 1−π_0_ of genes in a manner also described in the Methods section. What proportion of genes is selected may depend upon how many genes in an HDE are expected to be differentially expressed. This may determine whether a proportion equal to 0.01 or 0.5 is chosen to construct a plasmode. Proportions between 0.05 and 0.2 were used here as they are in the range of estimated proportions of differentially expressed genes that we have seen from the many data sets we have analyzed.

Finally, the plasmode data set was analyzed using a selected statistical method. We used two sample t-tests to obtain a plasmode distribution of P-values for each plasmode data set because the methods compared herein all analyze a distribution of P-values from an HDE. P-values were declared statistically significant if smaller than a threshold τ. Box 1 summarizes symbol definitions.

When comparing the 15 statistical methods, we used three values of π_0_ (0.8, 0.9, and 0.95) and two thresholds (τ = 0.01 and 0.001). For each choice of π_0_ and threshold τ, we ran B = 100 simulations. All 15 methods provided estimates of π_0_, 14 provided estimates of FDR, and 7 provided estimates of LFDR. Because the true values of π_0_ and FDR are known for each plasmode data set, we can compare the accuracy of estimates from the different methods.

### Methods for Estimating FDR

There are two basic strategies for estimating FDR, both predicated on an estimated value for π_0_, the first using equation (1) below, the second using a mixture model approach. Let *P_K_* = *M*/*K* be the proportion of tests that were declared significant at a given threshold, where *M* and *K* were defined with respect to quantities in Table 1. Then one estimate for *FDR* at this threshold is,
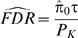
(1)The mixture model (usually a two-component mixture) approach uses a model of the form,
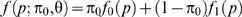
(2)where *f* is a density, *p* represents a P-value, *f*_0_ a density of a P-value under the null hypothesis, *f*_1_ a density of a P-value under the alternative hypothesis, π_0_ is interpreted as before, and θ a (possibly vector) parameter of the distribution. Since valid P-values are assumed, *f*_0_ is a uniform density. LFDR is defined with respect to this mixture model as,
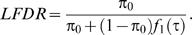
(3)FDR is defined similarly except that the densities in (3) are replaced by the corresponding cumulative distribution functions (CDF), that is,
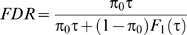
(4)where *F*_1_(τ) is the CDF under the alternative hypothesis, evaluated at a chosen threshold τ. (There are different definitions of FDR and the definition in (4) is, under some conditions, the definition of a positive false discovery rate [12]. However, in cases with a large number of genes many of the variants of FDR are very close [13]).

**Table 1:** Quantities of interest in microarray experiments.

	Genes for which there is not a real effect	Genes for which there is a real effect
Genes not declared significant at designated threshold	*A*	*B*
Genes declared significant at designated threshold	*C*	*D*

*A*+*B*+*C*+*D* = *K* = the number of genes analyzed in a microarray experiment. *M* = *C*+*D* is the number of rejected null hypotheses.

The methods are listed for quick reference in Table 2. Methods 1–8 use different estimates for π_0_ and, as implemented herein, proceed to estimate FDR using equation (1). Method 9 uses a unique algorithm to estimate LFDR and does not supply an estimate of FDR. Methods 10–15 are based on a mixture model framework and estimate FDR and LFDR using equations (3) and (4) where the model components are estimated using different techniques. All methods were implemented using tuning parameter settings from the respective paper or ones supplied as default values with the code in cases where the code was published online.

**Table 2:** Fifteen methods with the source of the software used herein.

Method	Citation	Source of code
1	Benjamini and Hochberg [6]	GeneTS
2	Benjamini and Hochberg [14]	GeneTS
3	Mosig et al., [15]	Website
4	Storey & Tibshirani [16]	Qvalue
5	Storey, Taylor, Siegmund [17]	Qvalue
6	Schweder and Spjøtvoll [18]	Coded by us
7	Dalmasso, Broët, and Moreau [19]	Author website
8	Langaas, Lindqvist, Ferkingstad [20]	Limma
9	Scheid and Spang [21]	Twilight
10	Pounds and Morris [22]	Author website
11	Pounds and Cheng [23]	Author website
12	Liao et al., [24]	Author website
13	Broberg [25]	SAGx
14	Broberg [25]	SAGx
15	Allison et al., [26]	From authors

Most software was available as an R library at www.r-project.org, and was otherwise available from an author's website or coded by us.

### Results of the Statistical Methods Tests

First, to compare their differences, we used the 15 methods to analyze the original two data sets, with data set 1 having a “stronger signal” (i.e., lower estimates of π_0_ and FDR). Estimates of π_0_ from methods 3 through 15 ranged from 0.742 to 0.837 for data set 1 and 0.852 to 0.933 for data set 2. (Methods 1 and 2 are designed to control for rather than estimate FDR and are designed to be conservative; hence, their estimates were much closer to 1.) Results of these analyses can be seen in the Supplementary Tables S1 and S2.

**Figure 3:**
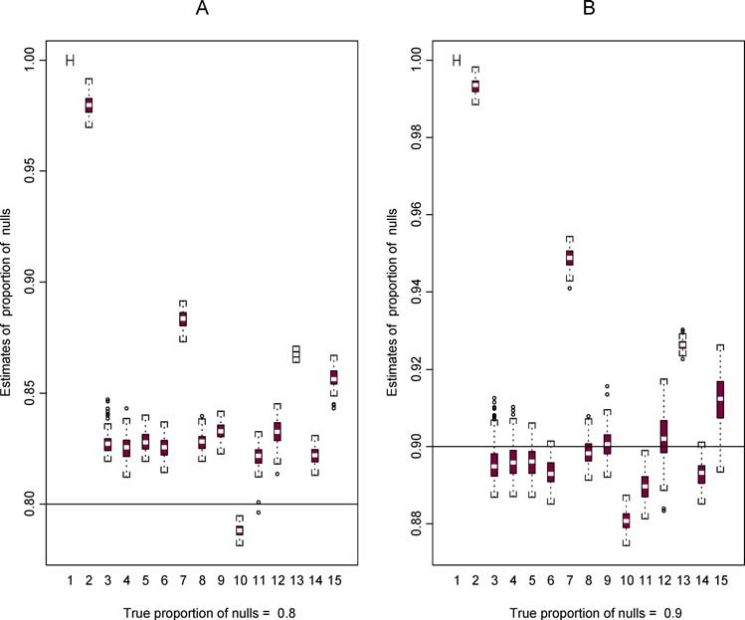
Boxplots for estimates of π_0_ from 100 plasmodes based on data set 2 for the 15 methods. Two cases are shown representing A. π_0_ = 0.8 and B. π_0_ = 0.9, represented by the horizontal line in the two plots A and B, respectively.

**Figure 4:**
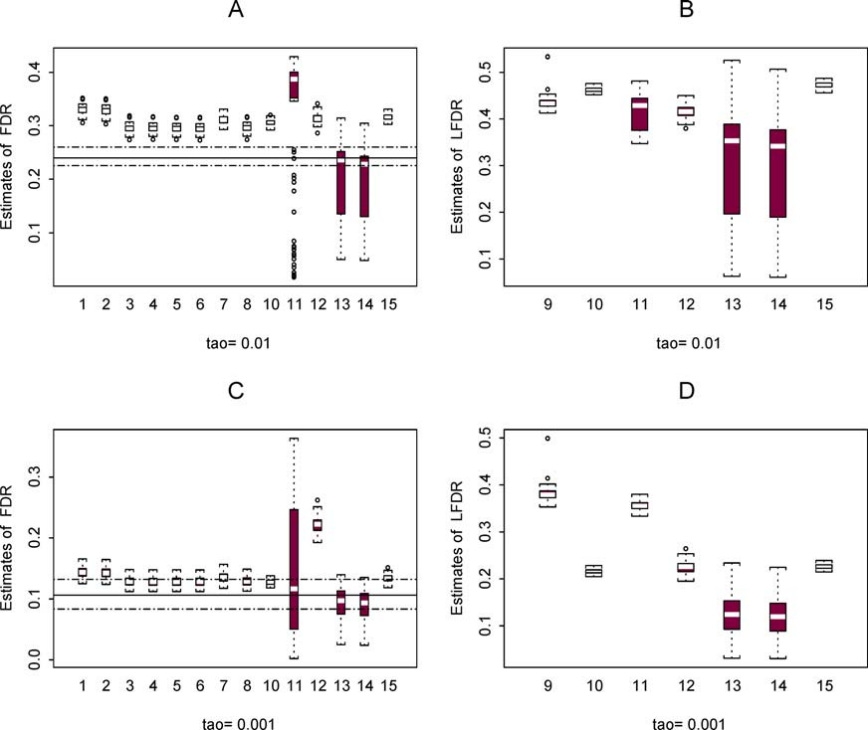
Plots of estimated *FDR* (A and C) and *LFDR* (B and D) using the 15 methods in 100 plasmodes from data set 2 for the case where π_0_ = 0.9. Estimates calculated at two thresholds τ = 0.01 (A and B) and 0.001 (C and D) are shown. For the plots of *FDR* estimates, the horizontal line is the mean of the 100 true values of *FDR* in the plasmodes and the horizontal dashed lines are the minimum and maximum. True values of *LFDR* are not known.

Next, using the two template data sets we constructed plasmode data sets in order to compare the performance of the 15 methods for estimating π_0_ (all methods), FDR (all methods except method 9), and LFDR (methods 9–15). Figures 3 and 4 show some results based on data set 2. More results are available in the Figures S1, S2, S3, S4, S5, and S6.

Figure 3 shows the distribution of 100 estimates for π_0_ using data set 2 when the true value of π_0_ is equal to 0.8 and 0.9. Methods 1 and 2 are designed to be conservative (i.e., true values are overestimated). With a few exceptions, the other methods tend to be conservative when π_0_ = 0.8 and liberal (the true value is underestimated) when π_0_ = 0.9. The variability of estimates for π_0_ is similar across methods, but some plots show a slightly larger variability for methods 12 and 15 when π_0_ = 0.9.

Figure 4 shows the distribution of estimates for FDR and LFDR at the two thresholds. The horizontal lines in the plots show the mean (solid line) and the minimum and maximum (dashed lines) of the true FDR value for the 100 simulations. A true value for LFDR is not known in the simulation procedure. The methods tend to be conservative (overestimate FDR) when the threshold τ = 0.01 and are more accurate at the lower threshold. Estimates of FDR are more variable for methods 11, 13, and 14 and estimates for LFDR more variable for methods 13 and 14, with the exception of a few unusual estimates obtained from method 9. The high variability of FDR estimates from method 11 may be due to a “less than optimal” choice of the spanning parameter in a numerical smoother (see also Pounds and Cheng [27]). We did not attempt to tune any of the methods for enhanced performance.

## Discussion

Researchers have been evaluating the performance of the burgeoning number of statistical methods for the analysis of high dimensional omic data, relying on a mixture of mathematical derivations, computer simulations, and sadly, often single dataset illustrations or mere *ipse dixit* assertions. Recognizing that the latter two approaches are simply unacceptable approaches to method validation [2] and that the first two suffer from limitations described earlier, an increasing number of investigators are turning to plasmode datasets for method evaluation [28]. An excellent example is the Affycomp website (http://affycomp.biostat.jhsph.edu/) that allows investigators to compare different microarray normalization methods on datasets of known structure. Other investigators have also recently used plasmode-like approaches which they refer to as ‘data perturbation’ [29],[30], yet it is not clear that these ‘perturbed datasets’ can distinguish true from false positives, suggesting greater need for articulation of principles or standards of plasmode generation.

As more high dimensional experiments with larger sample sizes become available, researchers can use a new kind of simulation experiment to evaluate the performance of statistical analysis methods, relying on actual data from previous experiments as a template for generating new data sets, referred to herein as plasmodes. In theory, the plasmode method outlined here will enable investigators to choose *on an empirical basis* the most appropriate statistical method for their HDEs.

Our results also suggest that large, searchable databases of plasmode data sets would help investigators find existing data sets relevant to their planned experiments. (We have already implemented a similar idea for planning sample size requirements in HDEs [31],[32].) Investigators could then use those data sets to compare and evaluate several analytical methods to determine which best identifies genes affected by the treatment condition. Or, investigators could use the plasmode approach on their own data sets to glean some understanding of how well a statistical method works on their type of data. Our results compare the performance of 15 statistical methods as they process the specific plasmode data sets constructed from the CNGI data. Although identifying one uniformly superior method (if there is one) is difficult within the limitations of this one comparison, our results suggest that certain methods could be sensitive to tuning parameters or different types of data sets. A comparison over multiple types of source data sets with different distributions of effects sizes could add the detail necessary to clearly recommend certain methods over others [1].

Other papers have used simulation studies to compare the performance of methods for estimating π_0_ and FDR (e.g., Hsueh et al. [33]; Nguyen [34]; Nettleton et al. [35]). We compared methods that use the distribution of P-values as was done in Broberg [36] and Yang and Yang [37]. Unlike our plasmode approach, most earlier comparison studies used normal distributions to simulate gene expression data and incorporated dependence using a block diagonal correlation structure as in Allison et al [26].

A key implication and recommendation of our paper is that, as data from the growing number of HDEs is made publicly available, researchers may identify a previous HDE similar to one they are planning or have recently conducted and use data from these experiments to construct plasmode data sets with which to evaluate candidate statistical methods. This will enable investigators to choose the most appropriate method(s) for analyzing their own data and thus to increase the reliability of their research results. In this manner, statistical science (as a discipline that studies the methods of statistics) becomes as much an empirical science as a theoretical one.

## Methods

The quantities in Table 1 are those for a typical microarray experiment. Let *N* = *A*+*B* and *M* = *C*+*D* and note that both *N* and *M* will be known and *K* = *N*+*M*. However, the number of false discoveries is equal to an unknown number *C*. The proportion of false discoveries for this experiment is *C/M*. Benjamini and Hochberg [6] defined *FDR* as, 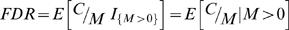 *P*(*M*>0) where *I*_{*M*>0}_ is an indicator function equal to 1 if *M*>0 and zero otherwise. Storey [12] defined the positive *FDR* as 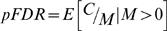. Since *P*(*M*>0)≥1−(1−τ)*^K^*, and since *K* is usually very large, *FDR*≈*pFDR*, so we do not distinguish between *FDR* and *pFDR* as the parameter being estimated and simply refer to it as *FDR* with estimates denoted 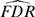 (and 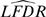).

Suppose we identify a template data set corresponding to a two treatment comparison for differential gene expression for *K* genes. Obtain a vector, δ, of effect sizes. One suggestion is the usual t-statistic, where the i^th^ component of δ, is given by
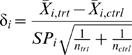
(5)where *n_trt_*, *n_ctrl_* are number of biological replicates in the treatment and control group, respectively, *X̅* *_i_*_,*trt*_, *X̅* *_i_*_,*ctrl*_ are the mean gene expression levels for gene *i* in treatment and control groups, and 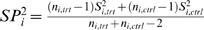, is the usual pooled sample variance for the ith gene, where the two sample variances are given by 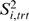, 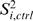. In what follows, we will use this choice for δ*_i_* since it allows for effects to be described by a unitless quantity, i.e., it is scaled by the standard error of the observed mean difference *X̅* *_i_*_,*trt*_−*X̅* *_i_*_,*ctrl*_ for each gene.

For convenience, assume that *n_ctrl_* is an even number and divide the control group into two sets of equal size. Requiring *n_ctrl_*≥4 allows for at least two arrays in each set, thus allowing estimates of variance within each of the two sets. This will be the basis for the plasmode “null” data set. There are 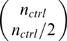 ways of making this division. Without loss of generality, assume that the first *n_ctrl_*/2 arrays after the division are the plasmode control group and the second *n_ctrl_*/2 are the plasmode treatment group. Specify a value of π_0_ and specify a threshold, τ, such that a P-value ≤τ is declared evidence of differential expression. Execute the following steps.

1. Sample without replacement (1−π_0_)*K* (rounding down to the nearest integer) from the integers 1,…, *K*. Denote this set as *S* ^*^. This set will denote those genes that will be differentially expressed.
2. Sample (1−π_0_)*K* (rounding down) effect sizes without replacement from the vector δ with components given by equation (5), where the ith component is selected with a weighted probability, 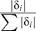. Denote this vector as δ ^*^. This will be the set of effect sizes used to differentially express genes. The weighted probability sampling allows for the fact that the original vector δ contains effects for both differentially expressed genes and genes corresponding to true null hypotheses. Thus larger effects are more likely to be selected, but the chance remains for very small effects to be selected as well. The weighted probabilities could be modified to allow for a higher (or lower) probability of large effects being sampled and, as such, could be a tuning adjustment in a plasmode simulation procedure.
3. For each expression level in the plasmode treatment group and for each gene, *j*, in the set *S* ^*^, add the amount 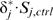 where *S_j,ctrl_* is the sample standard deviation for the *j*th gene in the original control group. This is one plasmode data set with a null reference data set obtained within the control group but effect sizes borrowed from the full microarray experiment.
4. Conduct a statistical test for differentially expressed genes on the plasmode data set and record the distribution of P-values. Determine which genes have P-values ≤τ.
5. Note that π_0_ and the set *S* ^*^ are known, so a true value of FDR for this data set is available. This true value will change with each simulated data set since the set *S* ^*^ and the vector δ ^*^ will be different in each simulation.
6. Apply a statistical method that estimates π_0_, FDR, LFDR and other quantities of interest. Estimates of FDR and LFDR are computed at a preset threshold τ. Some methods compute these estimates at the observed P-values in which case we interpolate the estimates computed at the two nearest P-values above and below τ.
7. Repeat steps 1–6 B times. Record summary statistics such as the mean, standard deviation, and range of the true FDR over the B plasmodes, and the summary statistics from the estimates obtained from the statistical method that is being evaluated.
8. Choose another threshold τ and/or another value of π_0_ and repeat for a new simulation case.

One can then obtain another data set and repeat the entire process to evaluate a method on a different type of data, perhaps from a different organism having a different null distribution, or a different treatment type giving a different distribution of effect sizes, δ. Alternatively, one might choose to randomly divide the control group again and repeat the entire process. This would help assess how differences in arrays within a group or possible correlation structure might affect results from a method. If some of the arrays in the control group have systematic differences among them (e.g., differences arising from variations in experimental conditions—day, operator, technology, etc.), then the null distribution can be sensitive to the random division of the original control group into the two plasmode groups, particularly if *n_ctrl_* is small.

**Box 1:** Notation for parameters used in modeling high dimensional data

π_0_ = A true proportion of genes for which there is no differential expression. This value is controlled by the experimenter in a simulation study.
1−π_0_ = the proportion of genes that are truly differentially expressed.
πˆ_0_ = An estimate of π_0_ obtained using a statistical method on data from an HDE.
τ = A threshold set by the investigator below which P-values are declared statistically significant.

## Supporting Information

Figure S1 Boxplots plasmode simulations dataset 1.
(0.02 MB PDF)

Figure S2 Boxplots plasmode simulations dataset 2.
(0.02 MB PDF)

Figure S3 Plots of FDR & LFDR dataset 1.
(0.03 MB PDF)

Figure S4 Plots of FDR & LFDR dataset 1 at 0.9.
(0.03 MB PDF)

Figure S5 Plots of FDR & LFDR dataset 2.
(0.02 MB PDF)

Figure S6 Plots of FDR & LFDR dataset 2 at 0.9.
(0.03 MB PDF)

Table S1 Methods comparison dataset 1.
(0.02 MB PDF)

Table S2 Methods comparison dataset 2.
(0.02 MB PDF)
