# Supplementary figures and images for: Evaluating Statistical Methods Using Plasmode Data Sets in the Age of Massive Public Databases: An Illustration Using False Discovery Rates
Source: PLoS Genet. 2008 Jun 20;4(6):e1000098. doi: 10.1371/journal.pgen.1000098 (PMC2409977; doi:10.1371/journal.pgen.1000098)

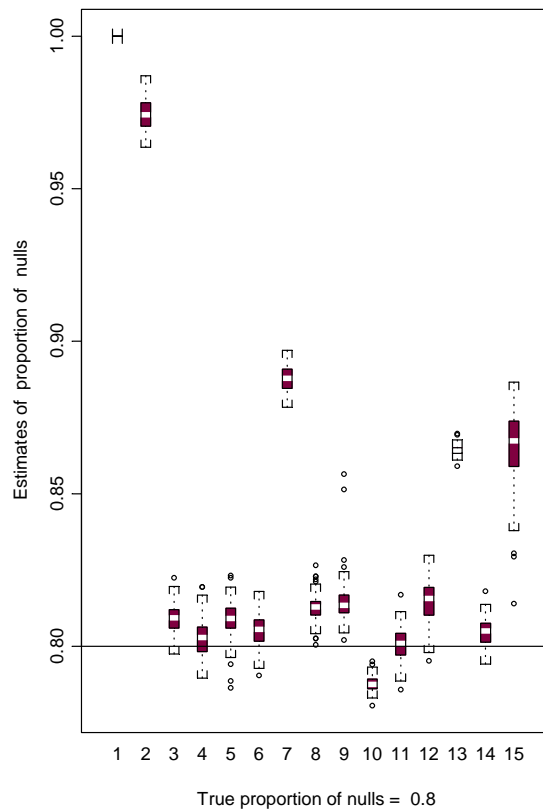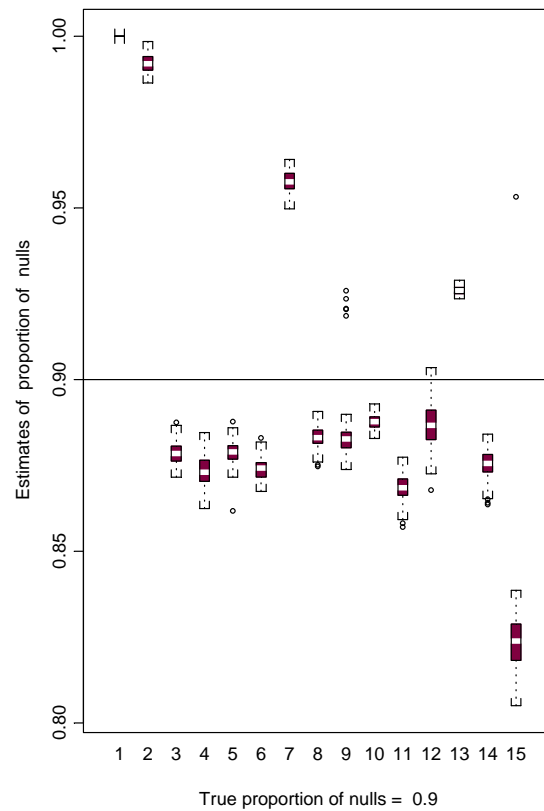

Supplement: Figure S1 — Boxplots plasmode simulations dataset 1. (0.02 MB PDF) [file pgen.1000098.s001.pdf]

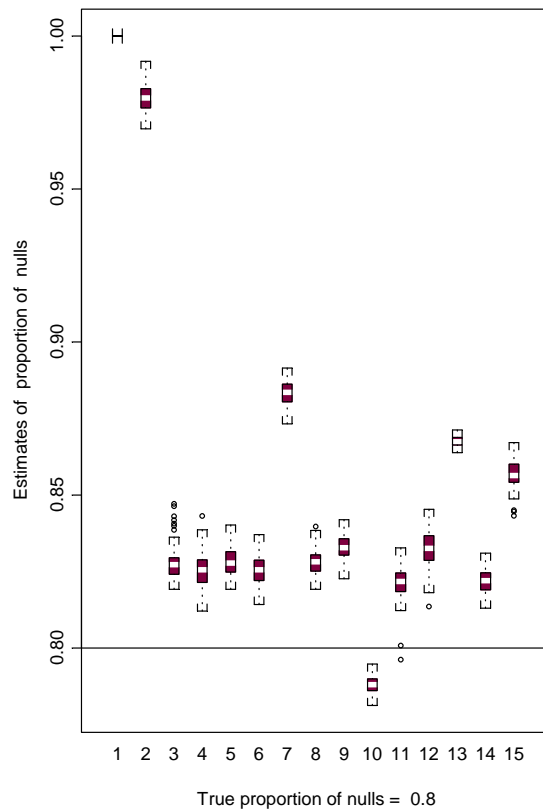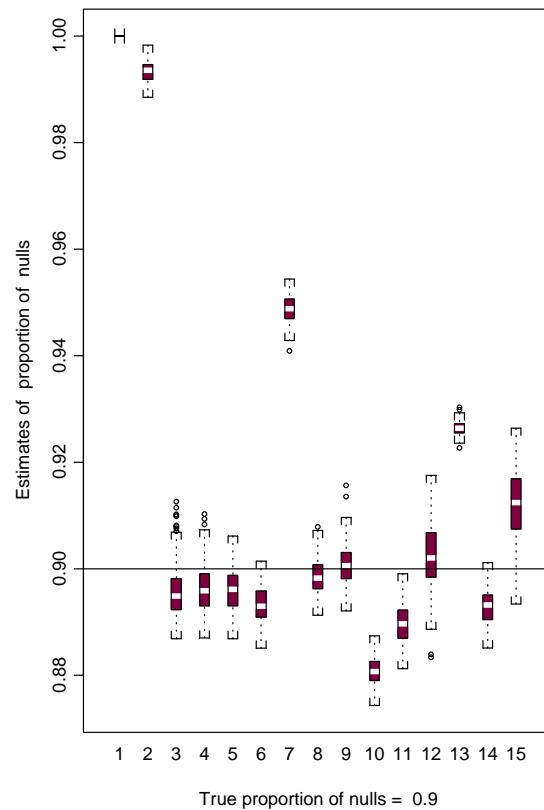

Supplement: Figure S2 — Boxplots plasmode simulations dataset 2. (0.02 MB PDF) [file pgen.1000098.s002.pdf]

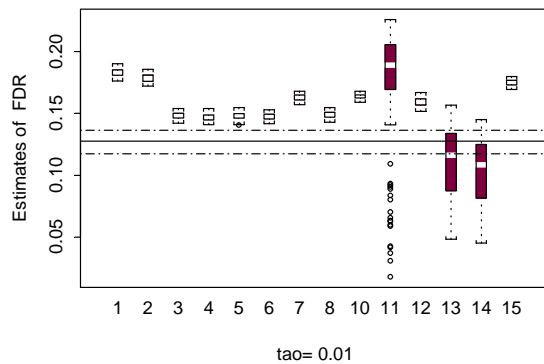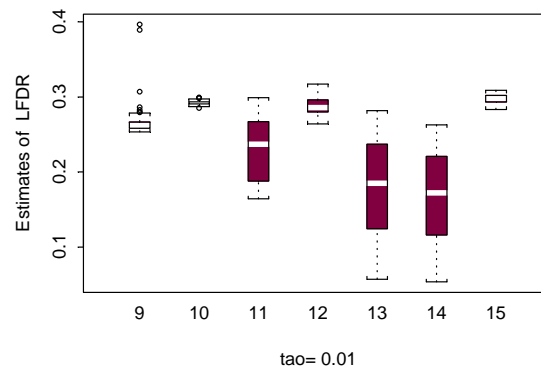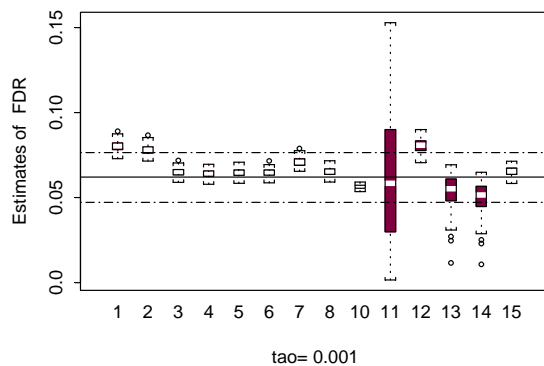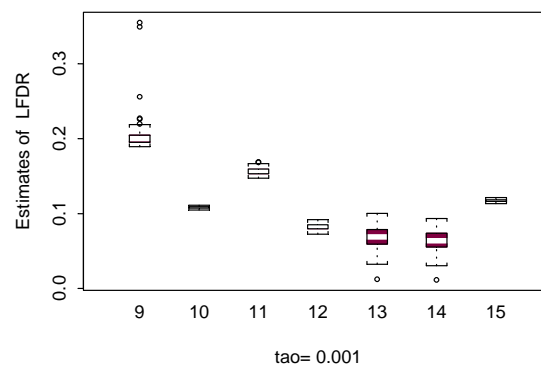

Supplement: Figure S3 — Plots of FDR & LFDR dataset 1. (0.03 MB PDF) [file pgen.1000098.s003.pdf]

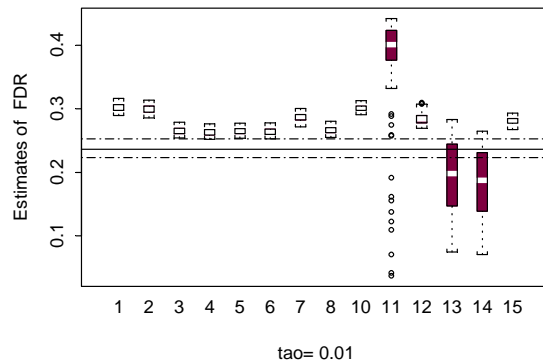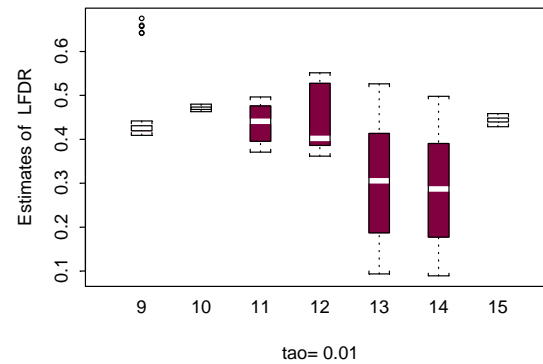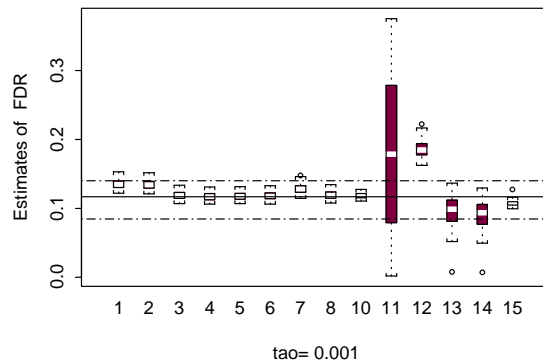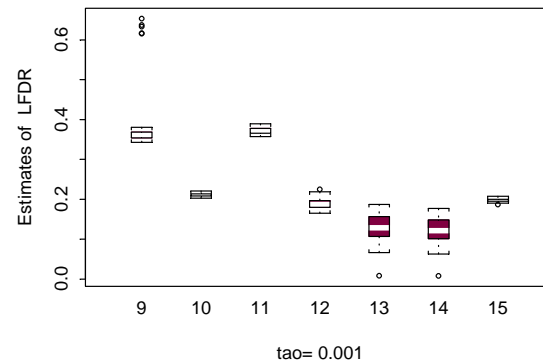

Supplement: Figure S4 — Plots of FDR & LFDR dataset 1 at 0.9. (0.03 MB PDF) [file pgen.1000098.s004.pdf]

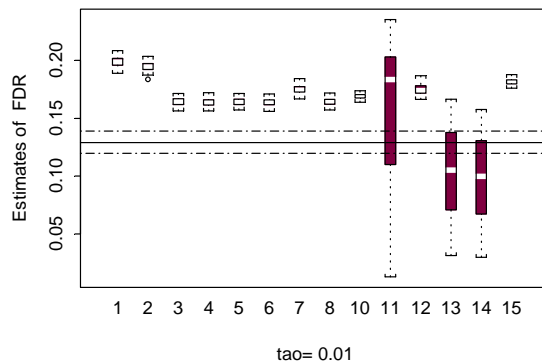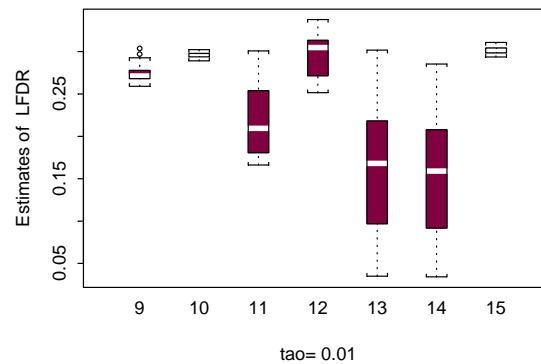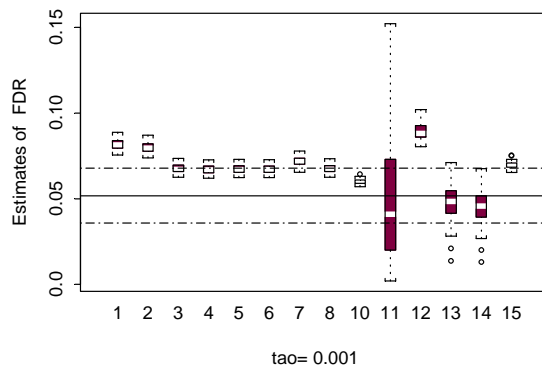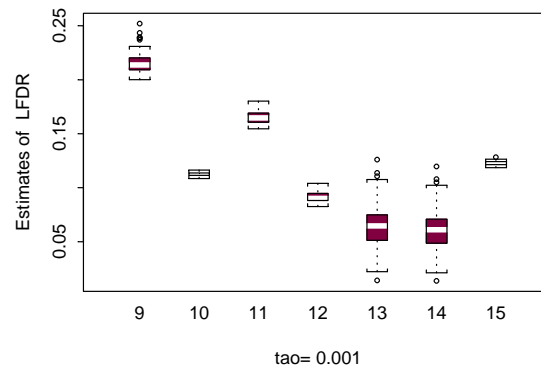

Supplement: Figure S5 — Plots of FDR & LFDR dataset 2. (0.02 MB PDF) [file pgen.1000098.s005.pdf]

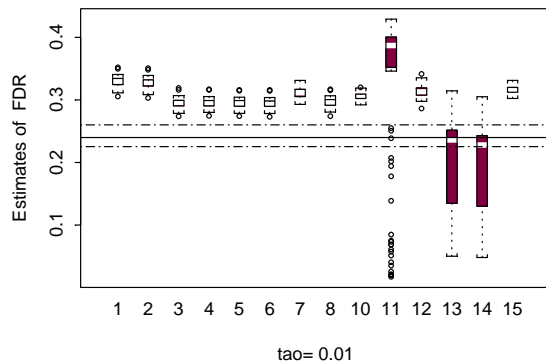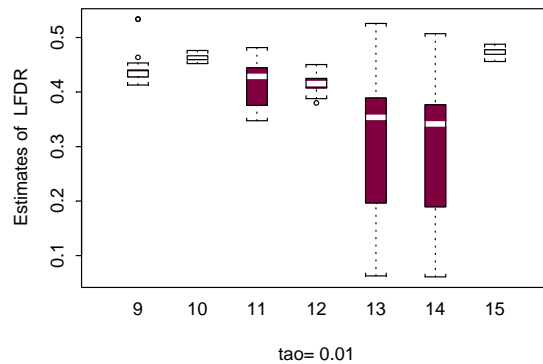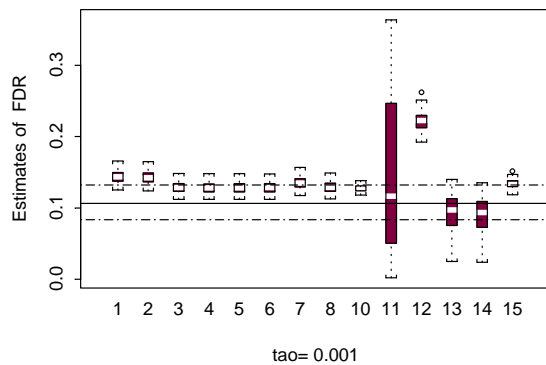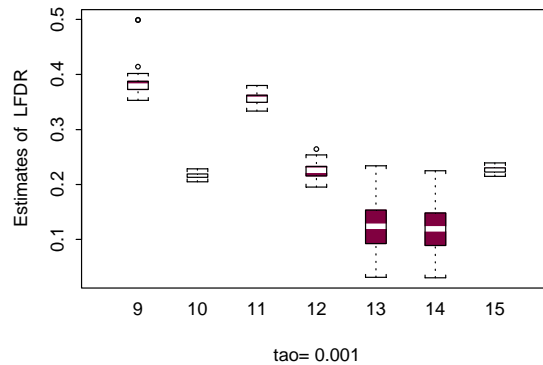

Supplement: Figure S6 — Plots of FDR & LFDR dataset 2 at 0.9. (0.03 MB PDF) [file pgen.1000098.s006.pdf]
