# Supplementary material for: Evaluating Statistical Methods Using Plasmode Data Sets in the Age of Massive Public Databases: An Illustration Using False Discovery Rates
Source: PLoS Genet. 2008 Jun 20;4(6):e1000098. doi: 10.1371/journal.pgen.1000098 (PMC2409977; doi:10.1371/journal.pgen.1000098)
Supplement: Table S1 — Methods comparison dataset 1. (0.02 MB PDF) [file pgen.1000098.s007.pdf]

Table S1: Comparison of methods for template data set 1 (Control versus EGCG treatment group for day 21) analyzed on the MASS 5 processed images. Estimates of π_0 and for FDR, and LFDR computed at two thresholds for those methods that provide estimates. Estimates are given as NA for those methods that do not produce an estimate for one or the other.

Method	$\hat{\pi}_0$	$\tau = 0.01$		$\tau = 0.001$	
		$\widehat{FDR}$	$\widehat{LFDR}$	$\widehat{FDR}$	$\widehat{LFDR}$
1 (BH)	1	0.197	NA	0.077	NA
2 (BH-A)	0.982	0.194	NA	0.076	NA
3 (M-L)	0.787	0.155	NA	0.061	NA
4 (ST-S)	0.781	0.154	NA	0.060	NA
5 (ST- B)	0.789	0.156	NA	0.061	NA
6 (P-plot)	0.786	0.155	NA	0.061	NA
7 (LBE)	0.826	0.163	NA	0.064	NA
8 (Convest)	0.789	0.156	NA	0.061	NA
9 (SEP)	0.7906	NA	0.238	NA	0.190
10 (BUM)	0.742	0.147	0.252	0.055	0.098
11(SPLOSH)	0.784	0.046	0.161	0.035	0.147
12 (L-L)	0.790	0.164	0.261	0.082	0.083
13 (MGF)	0.837	0.049	0.069	0.037	0.052
14 (PRE)	0.782	0.046	0.065	0.034	0.048
15(HDBMix)	0.801	0.160	0.265	0.062	0.108
