# Supplementary material for: Evaluating Statistical Methods Using Plasmode Data Sets in the Age of Massive Public Databases: An Illustration Using False Discovery Rates
Source: PLoS Genet. 2008 Jun 20;4(6):e1000098. doi: 10.1371/journal.pgen.1000098 (PMC2409977; doi:10.1371/journal.pgen.1000098)
Supplement: Table S2 — Methods comparison dataset 2. (0.02 MB PDF) [file pgen.1000098.s008.pdf]

Table S2: Comparison of methods for template data set 2 (Control versus Resveratrol treatment group for day 50) analyzed on the MASS 5 processed images. Table entries are those described in Table 1.

Method	$\hat{\pi}_0$	$\tau = 0.01$		$\tau = 0.001$	
		$\widehat{FDR}$	$\widehat{LFDR}$	$\widehat{FDR}$	$\widehat{LFDR}$
1 (BH)	1	0.711	NA	0.536	NA
2 (BH-A)	0.999	0.711	NA	0.536	NA
3 (M-L)	0.873	0.620	NA	0.468	NA
4 (ST-S)	0.870	0.614	NA	0.456	NA
5 (ST- B)	0.876	0.619	NA	0.459	NA
6 (P-plot)	0.878	0.624	NA	0.471	NA
7 (LBE)	0.933	0.659	NA	0.489	NA
8 (Convest)	0.870	0.618	NA	0.466	NA
9 (SEP)	0.881	NA	0.666	NA	0.660
10 (BUM)	1	1	1	1	1
11(SPLOSH)	0.852	0.597	0.605	0.517	0.589
12 (L-L)	0.855	0.645	0.648	0.642	0.642
13 (MGF)	0.924	0.587	0.684	0.477	0.515
14 (PRE)	0.865	0.550	0.640	0.446	0.482
15(HDBMix)	0.875	0.609	0.624	0.579	0.592
